# *Trans*-bis­(3-*tert*-butyl­pyridine-κ*N*)bis­(4-*tert*-butyl­pyridine-κ*N*)bis­(thio­cyanato-κ*N*)manganese(II)

**DOI:** 10.1107/S1600536812041128

**Published:** 2012-10-06

**Authors:** Thorben Reinert, Inke Jess, Christian Näther

**Affiliations:** a Institut für Anorganische Chemie, Christian-Albrechts-Universität Kiel, Max-Eyth Str. 2, D-24098 Kiel, Germany

## Abstract

The asymmetric unit of the title compound [Mn(NCS)_2_(C_9_H_13_N)_4_] consists of one Mn^II^ cation located on a center of inversion, one thio­cyanato anion, one 3-*tert*-butyl­pyridine ligand and one 4-*tert*-butyl­pyridine ligand in general positions. The *tert*-butyl group of the 4-*tert*-butyl­pyridine ligand is disordered over two sets of sites in a 0.60:0.40 ratio. The Mn^II^ cation is coordinated by six N atoms of four *tert*-butyl­pyridine ligands and two *N*-bonded thio­cyanato anions within a slightly distorted octa­hedral coordination environment.

## Related literature

For related structures, see: Nassimbeni *et al.* (1990 ▶) (4-*tert*-butyl­pyridine only). For the background to this work, see: Boeckmann & Näther (2010 ▶, 2011 ▶).
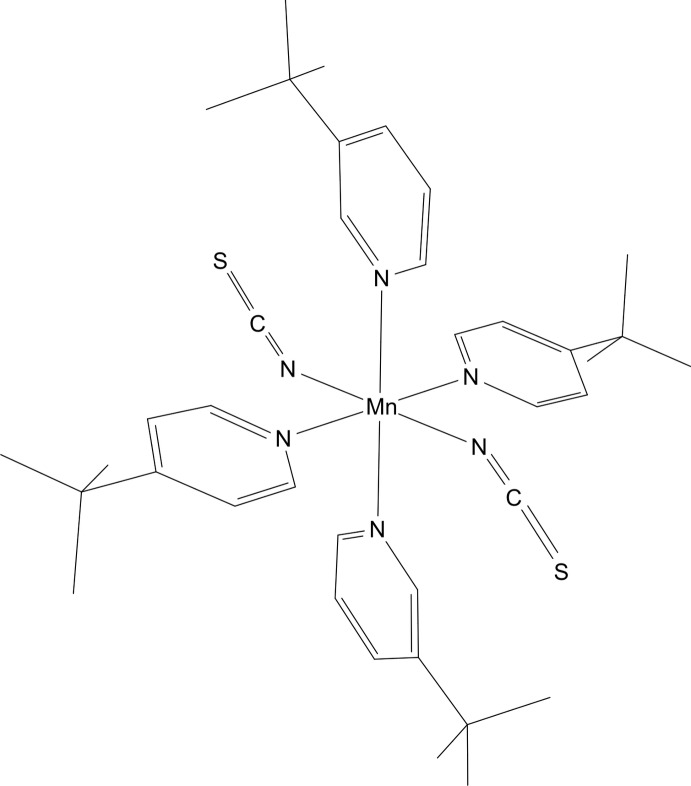


## Experimental

### 

#### Crystal data

[Mn(NCS)_2_(C_9_H_13_N)_4_]
*M* *_r_* = 711.92
Triclinic, 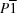

*a* = 9.5921 (7) Å
*b* = 10.7253 (9) Å
*c* = 11.6286 (10) Å
α = 66.870 (9)°
β = 68.011 (9)°
γ = 76.359 (9)°
*V* = 1014.59 (17) Å^3^
*Z* = 1
Mo *K*α radiation
μ = 0.46 mm^−1^
*T* = 200 K
0.13 × 0.09 × 0.05 mm

#### Data collection

STOE IPDS-1 diffractometer
7271 measured reflections
3845 independent reflections
3017 reflections with *I* > 2σ(*I*)
*R*_int_ = 0.037

#### Refinement

*R*[*F* ^2^ > 2σ(*F* ^2^)] = 0.049
*wR*(*F* ^2^) = 0.128
*S* = 1.03
3845 reflections
241 parameters
3 restraints
H-atom parameters constrained
Δρ_max_ = 0.92 e Å^−3^
Δρ_min_ = −0.88 e Å^−3^

### 

Data collection: *X-AREA* (Stoe, 2008 ▶); cell refinement: *X-AREA*; data reduction: *X-AREA*; program(s) used to solve structure: *SHELXS97* (Sheldrick, 2008 ▶); program(s) used to refine structure: *SHELXL97* (Sheldrick, 2008 ▶); molecular graphics: *XP* in *SHELXTL* (Sheldrick, 2008 ▶) and *DIAMOND* (Brandenburg, 2011 ▶); software used to prepare material for publication: *publCIF* (Westrip, 2010 ▶).

## Supplementary Material

Crystal structure: contains datablock(s) I, global. DOI: 10.1107/S1600536812041128/im2404sup1.cif

Structure factors: contains datablock(s) I. DOI: 10.1107/S1600536812041128/im2404Isup2.hkl

Additional supplementary materials:  crystallographic information; 3D view; checkCIF report

## Figures and Tables

**Fig. 1.**
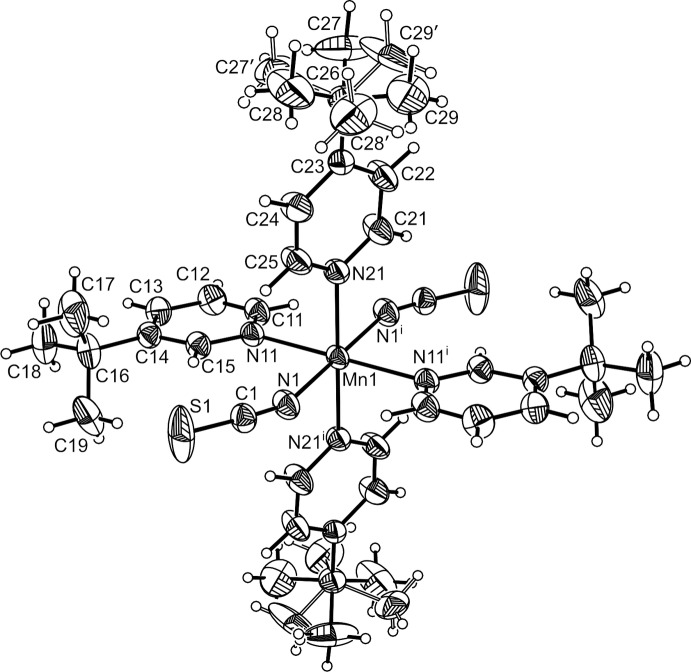
: Molecular structure of the title compund with displacement ellipsoids drawn at the 50% probability level (symmetry code: i = -x + 1, -y + 1, -z + 2). Disorder is shown as full and open bonds.

## supplementary crystallographic information

### Comment

The structure determination of the title compound was performed as part of a project on the synthesis of new coordination polymers based on transition metal thiocyanates and the investigations of their magnetic behaviour (Boeckmann *et al.*, 2010; Boeckmann *et al.*, 2011). Within this project we have reacted manganese(II)thiocyanate monohydrate with 4-*tert*-butylpyridine in water, which resulted in the formation of crystals of the title compound by accident. Apparently, the 4-*tert*-butylpyridine was contaminated with 3-*tert*-butylpyridine to a degree that allowed the formation of a few single crystals of the title compound. It was on the other hand not possible to obtain phase pure crystalline powder samples. In the crystal structure Mn atoms are surrounded by six N atoms of four *tert*-butylpyridine ligands and two *N*-bonded thiocyanato anions in mutual *trans* orientation in a slightly distorted octahedral geometry (Fig. 1). Mn···N distances range from 2.180 (3) Å to 2.337 (2) Å. It is also worth mentioning that so far no other compound containing 3-*tert*-butylpyridine has been reported in the CSD.

### Experimental

The title compound was obtained accidently during the reaction of 28.4 mg Mn(NCS)_2_ × H_2_O (0.15 mmol) with 44.4 µL 4-*tert*-butylpyridine (0.30 mmol), obtained from Sigma Aldrich, in 1.50 ml water at RT in a closed 3 mL snap cap vial. After three weeks colourless needles of the title compound were obtained.

### Refinement

H atoms were positioned with idealized geometry and were refined isotropically with *U*_iso_(H) = 1.2 *U*_eq_(C) (1.5 for methyl H atoms) of the parent atom using a riding model with C—H = 0.95 Å for aromatic and 0.98 Å for methyl hydrogen atoms. The *tert*-butyl group of the 4-*tert*-butylpyridine ligand is disordered and was refined using a split model with fixed site occupation factors of 0.60 and 0.40. Distances between the methyl groups in the two disordered moieties were restrained to be equal.

### Crystal data

**Table:**

[Mn(NCS)_2_(C_9_H_13_N)_4_]	*Z* = 1
*M**_r_* = 711.92	*F*(000) = 379
Triclinic, *P*1	*D*_x_ = 1.165 Mg m^−^^3^
Hall symbol: -P 1	Mo *K*α radiation, λ = 0.71073 Å
*a* = 9.5921 (7) Å	Cell parameters from 6934 reflections
*b* = 10.7253 (9) Å	θ = 1.9–28.2°
*c* = 11.6286 (10) Å	µ = 0.46 mm^−^^1^
α = 66.870 (9)°	*T* = 200 K
β = 68.011 (9)°	Needle, colourless
γ = 76.359 (9)°	0.13 × 0.09 × 0.05 mm
*V* = 1014.59 (17) Å^3^	

### Data collection

**Table:**

STOE IPDS-1 diffractometer	3017 reflections with *I* > 2σ(*I*)
Radiation source: fine-focus sealed tube	*R*_int_ = 0.037
Graphite monochromator	θ_max_ = 26.0°, θ_min_ = 2.5°
Phi scans	*h* = −11→11
7271 measured reflections	*k* = −13→12
3845 independent reflections	*l* = −14→14

### Refinement

**Table:**

Refinement on *F*^2^	Primary atom site location: structure-invariant direct methods
Least-squares matrix: full	Secondary atom site location: difference Fourier map
*R*[*F*^2^ > 2σ(*F*^2^)] = 0.049	Hydrogen site location: inferred from neighbouring sites
*wR*(*F*^2^) = 0.128	H-atom parameters constrained
*S* = 1.03	*w* = 1/[σ^2^(*F*_o_^2^) + (0.0656*P*)^2^ + 0.489*P*] where *P* = (*F*_o_^2^ + 2*F*_c_^2^)/3
3845 reflections	(Δ/σ)_max_ < 0.001
241 parameters	Δρ_max_ = 0.92 e Å^−^^3^
3 restraints	Δρ_min_ = −0.88 e Å^−^^3^

### Special details

**Table:**

Geometry. All e.s.d.'s (except the e.s.d. in the dihedral angle between two l.s. planes) are estimated using the full covariance matrix. The cell e.s.d.'s are taken into account individually in the estimation of e.s.d.'s in distances, angles and torsion angles; correlations between e.s.d.'s in cell parameters are only used when they are defined by crystal symmetry. An approximate (isotropic) treatment of cell e.s.d.'s is used for estimating e.s.d.'s involving l.s. planes.
Refinement. Refinement of *F*^2^ against ALL reflections. The weighted *R*-factor *wR* and goodness of fit *S* are based on *F*^2^, conventional *R*-factors *R* are based on *F*, with *F* set to zero for negative *F*^2^. The threshold expression of *F*^2^ > σ(*F*^2^) is used only for calculating *R*-factors(gt) *etc*. and is not relevant to the choice of reflections for refinement. *R*-factors based on *F*^2^ are statistically about twice as large as those based on *F*, and *R*- factors based on ALL data will be even larger.

### Fractional atomic coordinates and isotropic or equivalent isotropic displacement parameters (Å^2^)

**Table:**

	*x*	*y*	*z*	*U*_iso_*/*U*_eq_	Occ. (<1)
Mn1	0.5000	0.5000	1.0000	0.03151 (17)	
N1	0.6416 (3)	0.6660 (2)	0.9167 (2)	0.0419 (5)	
C1	0.6822 (3)	0.7724 (2)	0.8511 (2)	0.0383 (6)	
S1	0.73642 (11)	0.92047 (9)	0.75445 (13)	0.0912 (4)	
N11	0.3015 (2)	0.65601 (19)	0.94157 (18)	0.0352 (5)	
C11	0.1639 (3)	0.6132 (3)	0.9872 (2)	0.0418 (6)	
H11	0.1523	0.5200	1.0387	0.050*	
C12	0.0397 (3)	0.6983 (3)	0.9625 (3)	0.0481 (7)	
H12	−0.0555	0.6641	0.9950	0.058*	
C13	0.0547 (3)	0.8350 (3)	0.8892 (3)	0.0433 (6)	
H13	−0.0307	0.8950	0.8713	0.052*	
C14	0.1938 (3)	0.8843 (2)	0.8421 (2)	0.0367 (5)	
C15	0.3133 (3)	0.7888 (2)	0.8708 (2)	0.0370 (5)	
H15	0.4103	0.8199	0.8380	0.044*	
C16	0.2198 (3)	1.0344 (3)	0.7629 (3)	0.0493 (7)	
C17	0.3273 (5)	1.0455 (4)	0.6249 (3)	0.0840 (13)	
H17A	0.3451	1.1411	0.5740	0.126*	
H17B	0.2825	1.0116	0.5811	0.126*	
H17C	0.4235	0.9910	0.6310	0.126*	
C18	0.0701 (4)	1.1229 (3)	0.7584 (4)	0.0711 (10)	
H18A	0.0896	1.2182	0.7083	0.107*	
H18B	0.0043	1.1149	0.8483	0.107*	
H18C	0.0206	1.0922	0.7156	0.107*	
C19	0.2930 (4)	1.0855 (3)	0.8312 (4)	0.0690 (10)	
H19A	0.3097	1.1815	0.7815	0.103*	
H19B	0.3899	1.0312	0.8346	0.103*	
H19C	0.2258	1.0765	0.9210	0.103*	
N21	0.5965 (2)	0.4594 (2)	0.80246 (18)	0.0363 (5)	
C21	0.5945 (4)	0.3378 (3)	0.7974 (3)	0.0516 (7)	
H21	0.5477	0.2697	0.8769	0.062*	
C22	0.6561 (4)	0.3048 (3)	0.6840 (3)	0.0527 (8)	
H22	0.6509	0.2160	0.6872	0.063*	
C23	0.7253 (3)	0.3997 (2)	0.5657 (2)	0.0349 (5)	
C24	0.7275 (4)	0.5254 (3)	0.5714 (2)	0.0540 (8)	
H24	0.7735	0.5954	0.4934	0.065*	
C25	0.6638 (4)	0.5507 (3)	0.6890 (2)	0.0512 (7)	
H25	0.6684	0.6383	0.6888	0.061*	
C26	0.7927 (3)	0.3670 (3)	0.4375 (2)	0.0448 (6)	
C27	0.6680 (9)	0.3097 (12)	0.4244 (8)	0.100 (3)	0.60
H27A	0.6334	0.2307	0.5030	0.150*	0.60
H27B	0.7087	0.2819	0.3462	0.150*	0.60
H27C	0.5828	0.3802	0.4156	0.150*	0.60
C28	0.8526 (15)	0.4830 (7)	0.3205 (6)	0.113 (4)	0.60
H28A	0.8932	0.4547	0.2426	0.170*	0.60
H28B	0.9333	0.5145	0.3314	0.170*	0.60
H28C	0.7714	0.5572	0.3093	0.170*	0.60
C29	0.9189 (9)	0.2494 (8)	0.4536 (6)	0.084 (2)	0.60
H29A	0.8789	0.1724	0.5324	0.125*	0.60
H29B	1.0016	0.2798	0.4628	0.125*	0.60
H29C	0.9567	0.2210	0.3759	0.125*	0.60
C27'	0.7316 (12)	0.4873 (11)	0.3296 (8)	0.064 (3)	0.40
H27D	0.7487	0.5748	0.3277	0.096*	0.40
H27E	0.6231	0.4843	0.3513	0.096*	0.40
H27F	0.7852	0.4773	0.2431	0.096*	0.40
C28'	0.9667 (10)	0.3858 (13)	0.3845 (9)	0.079 (3)	0.40
H28D	0.9795	0.4746	0.3828	0.118*	0.40
H28E	1.0105	0.3812	0.2952	0.118*	0.40
H28F	1.0181	0.3132	0.4426	0.118*	0.40
C29'	0.769 (2)	0.2383 (12)	0.4445 (10)	0.116 (7)	0.40
H29D	0.8157	0.2285	0.3574	0.174*	0.40
H29E	0.6597	0.2319	0.4733	0.174*	0.40
H29F	0.8134	0.1657	0.5076	0.174*	0.40

### Atomic displacement parameters (Å^2^)

**Table:**

	*U*^11^	*U*^22^	*U*^33^	*U*^12^	*U*^13^	*U*^23^
Mn1	0.0411 (3)	0.0235 (3)	0.0266 (3)	−0.0085 (2)	−0.0061 (2)	−0.00652 (18)
N1	0.0488 (13)	0.0310 (11)	0.0420 (11)	−0.0114 (9)	−0.0091 (9)	−0.0093 (9)
C1	0.0349 (14)	0.0326 (13)	0.0469 (14)	−0.0053 (10)	−0.0150 (11)	−0.0099 (11)
S1	0.0620 (6)	0.0369 (4)	0.1414 (10)	−0.0216 (4)	−0.0427 (6)	0.0232 (5)
N11	0.0437 (12)	0.0289 (10)	0.0315 (10)	−0.0072 (9)	−0.0101 (8)	−0.0083 (8)
C11	0.0470 (15)	0.0324 (12)	0.0417 (13)	−0.0122 (11)	−0.0088 (11)	−0.0086 (10)
C12	0.0404 (15)	0.0459 (15)	0.0531 (15)	−0.0123 (12)	−0.0083 (12)	−0.0137 (12)
C13	0.0407 (15)	0.0400 (14)	0.0451 (14)	−0.0019 (11)	−0.0118 (11)	−0.0133 (11)
C14	0.0417 (14)	0.0324 (12)	0.0315 (11)	−0.0043 (10)	−0.0077 (10)	−0.0095 (9)
C15	0.0393 (14)	0.0326 (12)	0.0340 (12)	−0.0086 (10)	−0.0065 (10)	−0.0080 (9)
C16	0.0475 (16)	0.0301 (13)	0.0551 (16)	−0.0028 (11)	−0.0099 (13)	−0.0055 (11)
C17	0.109 (3)	0.0498 (19)	0.0509 (19)	−0.018 (2)	0.0052 (19)	0.0024 (15)
C18	0.066 (2)	0.0398 (16)	0.091 (2)	−0.0001 (15)	−0.0324 (19)	−0.0019 (16)
C19	0.062 (2)	0.0373 (16)	0.107 (3)	−0.0050 (14)	−0.0248 (19)	−0.0252 (17)
N21	0.0482 (13)	0.0308 (10)	0.0283 (9)	−0.0064 (9)	−0.0089 (8)	−0.0101 (8)
C21	0.078 (2)	0.0334 (13)	0.0322 (12)	−0.0183 (13)	0.0019 (12)	−0.0103 (10)
C22	0.079 (2)	0.0310 (13)	0.0393 (14)	−0.0149 (13)	−0.0011 (13)	−0.0138 (11)
C23	0.0412 (14)	0.0330 (12)	0.0311 (11)	−0.0033 (10)	−0.0115 (10)	−0.0116 (9)
C24	0.086 (2)	0.0397 (14)	0.0294 (12)	−0.0248 (14)	−0.0041 (13)	−0.0078 (11)
C25	0.084 (2)	0.0333 (13)	0.0350 (13)	−0.0208 (13)	−0.0081 (13)	−0.0116 (11)
C26	0.0581 (17)	0.0444 (14)	0.0312 (12)	−0.0050 (12)	−0.0094 (11)	−0.0166 (11)
C27	0.078 (5)	0.181 (10)	0.091 (5)	−0.010 (5)	−0.023 (4)	−0.102 (7)
C28	0.221 (12)	0.065 (4)	0.027 (3)	−0.048 (6)	0.011 (5)	−0.017 (3)
C29	0.082 (5)	0.098 (5)	0.063 (4)	0.024 (4)	−0.012 (3)	−0.048 (4)
C27'	0.079 (6)	0.082 (6)	0.036 (4)	−0.001 (5)	−0.020 (4)	−0.027 (4)
C28'	0.056 (5)	0.114 (8)	0.057 (5)	0.015 (5)	−0.006 (4)	−0.044 (6)
C29'	0.205 (17)	0.079 (7)	0.052 (6)	−0.083 (10)	0.040 (8)	−0.047 (6)

### Geometric parameters (Å, º)

**Table:**

Mn1—N1	2.180 (2)	C21—C22	1.378 (4)
Mn1—N1^i^	2.180 (2)	C21—H21	0.9500
Mn1—N21	2.3081 (18)	C22—C23	1.380 (3)
Mn1—N21^i^	2.3081 (18)	C22—H22	0.9500
Mn1—N11^i^	2.337 (2)	C23—C24	1.381 (4)
Mn1—N11	2.337 (2)	C23—C26	1.531 (3)
N1—C1	1.157 (3)	C24—C25	1.380 (4)
C1—S1	1.614 (3)	C24—H24	0.9500
N11—C11	1.343 (3)	C25—H25	0.9500
N11—C15	1.344 (3)	C26—C29'	1.419 (9)
C11—C12	1.368 (4)	C26—C28	1.467 (7)
C11—H11	0.9500	C26—C29	1.534 (7)
C12—C13	1.387 (4)	C26—C27	1.546 (8)
C12—H12	0.9500	C26—C28'	1.580 (10)
C13—C14	1.385 (4)	C26—C27'	1.583 (9)
C13—H13	0.9500	C27—H27A	0.9800
C14—C15	1.393 (4)	C27—H27B	0.9800
C14—C16	1.535 (3)	C27—H27C	0.9800
C15—H15	0.9500	C28—H28A	0.9800
C16—C17	1.524 (4)	C28—H28B	0.9800
C16—C18	1.530 (4)	C28—H28C	0.9800
C16—C19	1.537 (5)	C29—H29A	0.9800
C17—H17A	0.9800	C29—H29B	0.9800
C17—H17B	0.9800	C29—H29C	0.9800
C17—H17C	0.9800	C27'—H27D	0.9800
C18—H18A	0.9800	C27'—H27E	0.9800
C18—H18B	0.9800	C27'—H27F	0.9800
C18—H18C	0.9800	C28'—H28D	0.9800
C19—H19A	0.9800	C28'—H28E	0.9800
C19—H19B	0.9800	C28'—H28F	0.9800
C19—H19C	0.9800	C29'—H29D	0.9800
N21—C25	1.327 (3)	C29'—H29E	0.9800
N21—C21	1.333 (3)	C29'—H29F	0.9800
			
N1—Mn1—N1^i^	180.000 (1)	C23—C22—H22	119.8
N1—Mn1—N21	89.88 (8)	C22—C23—C24	115.3 (2)
N1^i^—Mn1—N21	90.12 (8)	C22—C23—C26	121.8 (2)
N1—Mn1—N21^i^	90.12 (8)	C24—C23—C26	122.9 (2)
N1^i^—Mn1—N21^i^	89.88 (8)	C25—C24—C23	120.9 (2)
N21—Mn1—N21^i^	180.000 (1)	C25—C24—H24	119.6
N1—Mn1—N11^i^	90.23 (8)	C23—C24—H24	119.6
N1^i^—Mn1—N11^i^	89.77 (8)	N21—C25—C24	123.6 (2)
N21—Mn1—N11^i^	86.16 (7)	N21—C25—H25	118.2
N21^i^—Mn1—N11^i^	93.84 (7)	C24—C25—H25	118.2
N1—Mn1—N11	89.77 (8)	C29'—C26—C28	128.7 (5)
N1^i^—Mn1—N11	90.23 (8)	C29'—C26—C23	116.5 (4)
N21—Mn1—N11	93.84 (7)	C28—C26—C23	114.0 (3)
N21^i^—Mn1—N11	86.16 (7)	C29'—C26—C29	61.8 (8)
N11^i^—Mn1—N11	180.00 (10)	C28—C26—C29	109.6 (6)
C1—N1—Mn1	157.6 (2)	C23—C26—C29	108.3 (3)
N1—C1—S1	177.4 (2)	C29'—C26—C27	44.1 (8)
C11—N11—C15	117.1 (2)	C28—C26—C27	111.9 (6)
C11—N11—Mn1	118.51 (16)	C23—C26—C27	106.9 (3)
C15—N11—Mn1	124.28 (17)	C29—C26—C27	105.7 (5)
N11—C11—C12	122.7 (2)	C29'—C26—C28'	111.6 (8)
N11—C11—H11	118.7	C28—C26—C28'	59.6 (6)
C12—C11—H11	118.7	C23—C26—C28'	107.0 (4)
C11—C12—C13	119.2 (3)	C29—C26—C28'	55.6 (5)
C11—C12—H12	120.4	C27—C26—C28'	145.3 (5)
C13—C12—H12	120.4	C29'—C26—C27'	111.2 (8)
C14—C13—C12	120.2 (3)	C28—C26—C27'	42.7 (5)
C14—C13—H13	119.9	C23—C26—C27'	107.4 (4)
C12—C13—H13	119.9	C29—C26—C27'	142.3 (4)
C13—C14—C15	116.1 (2)	C27—C26—C27'	74.8 (6)
C13—C14—C16	123.6 (2)	C28'—C26—C27'	102.1 (6)
C15—C14—C16	120.3 (2)	C26—C27—H27A	109.5
N11—C15—C14	124.7 (2)	C26—C27—H27B	109.5
N11—C15—H15	117.6	H27A—C27—H27B	109.5
C14—C15—H15	117.6	C26—C27—H27C	109.5
C17—C16—C18	111.4 (3)	H27A—C27—H27C	109.5
C17—C16—C14	109.0 (2)	H27B—C27—H27C	109.5
C18—C16—C14	111.0 (2)	C26—C28—H28A	109.5
C17—C16—C19	108.8 (3)	C26—C28—H28B	109.5
C18—C16—C19	107.7 (3)	H28A—C28—H28B	109.5
C14—C16—C19	108.8 (2)	C26—C28—H28C	109.5
C16—C17—H17A	109.5	H28A—C28—H28C	109.5
C16—C17—H17B	109.5	H28B—C28—H28C	109.5
H17A—C17—H17B	109.5	C26—C29—H29A	109.5
C16—C17—H17C	109.5	C26—C29—H29B	109.5
H17A—C17—H17C	109.5	H29A—C29—H29B	109.5
H17B—C17—H17C	109.5	C26—C29—H29C	109.5
C16—C18—H18A	109.5	H29A—C29—H29C	109.5
C16—C18—H18B	109.5	H29B—C29—H29C	109.5
H18A—C18—H18B	109.5	C26—C27'—H27D	109.5
C16—C18—H18C	109.5	C26—C27'—H27E	109.5
H18A—C18—H18C	109.5	H27D—C27'—H27E	109.5
H18B—C18—H18C	109.5	C26—C27'—H27F	109.5
C16—C19—H19A	109.5	H27D—C27'—H27F	109.5
C16—C19—H19B	109.5	H27E—C27'—H27F	109.5
H19A—C19—H19B	109.5	C26—C28'—H28D	109.5
C16—C19—H19C	109.5	C26—C28'—H28E	109.5
H19A—C19—H19C	109.5	H28D—C28'—H28E	109.5
H19B—C19—H19C	109.5	C26—C28'—H28F	109.5
C25—N21—C21	115.7 (2)	H28D—C28'—H28F	109.5
C25—N21—Mn1	123.21 (16)	H28E—C28'—H28F	109.5
C21—N21—Mn1	121.01 (15)	C26—C29'—H29D	109.5
N21—C21—C22	124.0 (2)	C26—C29'—H29E	109.5
N21—C21—H21	118.0	H29D—C29'—H29E	109.5
C22—C21—H21	118.0	C26—C29'—H29F	109.5
C21—C22—C23	120.5 (2)	H29D—C29'—H29F	109.5
C21—C22—H22	119.8	H29E—C29'—H29F	109.5

Symmetry code: (i) −*x*+1, −*y*+1, −*z*+2.
